# Screening of Antimicrobial Properties and Bioactive Compounds of *Pleurotus Ostreatus* Extracts against *Staphylococcus Aureus, Escherichia coli,* and *Neisseria Gonorrhoeae*

**DOI:** 10.1155/2023/1777039

**Published:** 2023-04-17

**Authors:** Sinethemba H. Yakobi, Senzosenkosi Mkhize, Ofentse J. Pooe

**Affiliations:** School of Life Sciences, Biochemistry, University of KwaZulu-Natal, Durban, South Africa

## Abstract

In recent years, the potential of pathogenic bacteria to acquire resistance to a variety of antimicrobial drugs has developed significantly due to the indiscriminate exposure of a number of antibiotic compounds. The purpose of this study is to determine the antibacterial capabilities and activities of crude *Pleurotus ostreatus* extracts against *Staphylococcus aureus* (ATCC 25923), *Escherichia coli* (ATCC 25922), *Neisseria gonorrhoeae* (ATCC 49926), and nine multidrug-resistant clinical isolates of *Neisseria gonorrhoeae*. All of these isolates exhibited sensitivity to azithromycin and ceftriaxone, while the majority of antibiotic resistance was seen against penicillin G, sulphonamide, and ciprofloxacin. Fifty percent of the isolates exhibited absolute resistance to both sulphonamide and ciprofloxacin, whereas 40% of the isolates displayed absolute resistance to penicillin G. The antibacterial activity of *P. ostreatus* extracts examined in this investigation varied within the same species of microorganisms. Extract *B* and *D*, extracted in the presence of 20% wheat bran bagasse and 20% maize flour bagasse, respectively, had exceptional antibacterial activity against all target isolates examined. We observed the lowest concentration of antibacterial agent required to inhibit the target bacteria to be between 1 × 10^−3^ mg/ml and 1 × 10^−6^ mg/ml with an estimated probability of 0.30769, a lower 95% confidence interval (CI) of 0.126807, an upper 95% CI of 0.576307, an estimated probability of 0.15385, a lower 95% CI of 0.043258, and an upper 95% CI, respectively. The MBC of 1 × 10^−3^ mg/ml was seen to eliminate 31% of the target bacteria. This dose was the most inhibitive. The antibacterial activity of all the extracts examined in the current study exhibited some degree of efficacy against both clinical isolates and standard strains. However, the majority of clinically isolated bacteria exhibited greater resistance to the extracts.

## 1. Introduction

Mushrooms have been shown to possess a variety of nutritional and nutraceutical properties and are a source of beneficial bioactive compounds [1]. Several preliminary studies have shown that some nutraceutical mushrooms have important cardioprotective, anticancer, antiviral, antibacterial, antiparasitic, anti-inflammatory, and antidiabetic properties [2, 3]. The *Pleurotus ostreatus* (*P. ostreatus*) mushroom is known to have pharmacologically active properties involved in several cellular mechanisms [4]. Despite this, the substrates employed in the mushroom extraction process have a substantial effect on the chemical and functional properties of the extract [5]. The antitumorigenic, immunomodulatory, antioxidant, cardiovascular, hypolipidemic, detoxifying, hepatoprotective, and antibacterial properties of *P. ostreatus* have attracted a great deal of attention from researchers over the last several years [6]. It has been shown that *P. ostreatus* mushrooms contain a vast array of unique bioactive chemicals. It is known that the solvents or substrates selected to extract these bioactive compounds have an influence not only on the class of bioactive compounds contained in the final extract but also on the overall quantity of the physicochemical properties of the compounds [7–9]. This, in turn, influences the range of pharmacological effects that these chemicals possess, including their capacity to limit bacterial growth [10]. Commonly, aqueous extraction is used in the process of assessing antibacterial potential [11]. Since aromatic and saturated organic compounds have been found to be the most potent antibacterial agents, methanol or ethanol is often used to extract them [11, 12]. According to the findings of a number of studies, mushroom extracts are a much more effective treatment for gram-positive bacteria than antibiotics [13]. As a direct consequence of the widespread incidence of microbial resistance, several antimicrobial agents have lost their ability to treat infectious diseases effectively [14] and the rapid appearance, selection, and dissemination of antibiotic-resistant bacteria necessitate the quest for novel MDR (multidrug resistance) infection treatment techniques [15]. Consequently, the development of alternative antimicrobial drugs operating via novel pathways remains an essential priority, and despite the countless efforts made in the quest for new treatment methods against multidrug-resistant illnesses, this objective has not yet been met [15, 16]. It appears that research has hit a brick wall in terms of identifying new classes of antibiotics and/or their chemical derivatives on which to base new therapies because the development and implementation of a new antimicrobial drug is difficult, time-consuming, and extremely expensive, and bacterial abilities to evolve resistance mechanisms are rapid and essentially limitless [17, 18]. Finding and exploiting natural compounds that may enhance the antibacterial effect of conventional antibiotics is a feasible approach in the ongoing battle against multidrug-resistant bacterial pathogens [19]. Studies have shown that the combination of naturally occurring compounds derived from mushrooms and regularly used antibacterial drugs may represent a revolutionary strategy against illnesses brought on by bacteria that are multidrug-resistant [20, 21]. Mushroom-derived polyphenolic compounds, such as flavonoids or phenolic acids, have been shown to have antimicrobial properties against a variety of microorganisms, are capable of making multidrug-resistant strains susceptible to bactericidal or bacteriostatic antibiotics, and are therefore promising natural antimicrobial agents [3, 13, 22]. More research into bioactive compounds derived from natural sources such as bacteria, fungi, and plants that are effective for treating pathogenic bacteria that are resistant to present treatment drugs would be very advantageous [23]. Studies show that mushroom species generate numerous bioactive compounds, including p-OH-phenylacetic acid, chlorogenic acid, ferulic acid, resveratrol, and chrysin. [24, 25]. However, researchers have not yet discovered the overwhelming majority of potentially bioactive molecules and vital natural components that may be present in *P. ostreatus* mushrooms [25, 26]. According to a recent study, *P. ostreatus* extract had a wide range of potential medical properties [27]. Based on the analysis, it was determined that *P. ostreatus* methanolic extracts have broad-spectrum antibacterial activity [28]. As a consequence, the prospect of producing antimicrobials from it seemed promising. Few other studies report on the chemical composition of *P. ostreatus*, and similar species have been released. The nutritional benefits of mushrooms have often been shown in dried fruit bodies [1]. It is said that fresh *Pleurotus* mushrooms typically have between 85% and 95% moisture content, and about 100 distinct bioactive substances may be found in the *P. ostreatus* fruiting body, which is primarily thought of as a possible new source of dietary fibre [29, 30]. While the nonstarch polysaccharides found in the cell walls of fungi, such as glucan, are abundant, phenolic compounds such as protocatechuic acid, gallic acid, homogentisic acid, rutin, myrictin, chrysin, and naringin, as are tocopherols like -tocopherol and -tocopherol, ascorbic acid, and carotene [31, 32]. They are also nutritious meals that are high in protein, lipids, carbs, vitamins, and minerals yet low in calories and fat [27]. It is now known that the *P. ostreatus* mushroom extracts contain important bioactive compounds and also function in a wide range of biological activities [8]. However, this can be compromised by the substrates applied in the mushroom extraction process. The objective of this study is to determine the effect of *P. ostreatus* extraction process on yield, productivity, bioactive compounds, and antibacterial activity against *Staphylococcus aureus* (ATCC 25923), *Escherichia coli* (ATCC 25922), *Neisseria gonorrhoeae* (ATCC 49926), and nine clinical isolates of *N. gonorrhoeae*.

## 2. Methods and Design

### 2.1. Preparation of Test Organisms

*Staphylococcus aureus* (ATCC 25923), *E. coli* (ATCC 25922), *and N. gonorrhoeae* (ATCC 49926), and nine clinical isolates of *N. gonorrhoeae* were chosen as the target bacteria. The target bacteria grew in 5 mL of Mueller-Hinton broth at 37°C for 18–24 hours. The amount of each test organism's inoculum was altered in order to achieve 1.5 × 10^8^ CFU/mL (0.5 McFarland) [33].

### 2.2. Preparation of *Pleurotus ostreatus* Mushrooms

*P. ostreatus* was grown on sugar cane bagasse and was grown in four steps: precultivation on PDA, spawn preparation, substrate preparation and inoculation, and fruiting. The test *P. ostreatus* mushroom was collected from Cedara College of Agriculture in Pietermaritzburg, KwaZulu Natal, South Africa. *P. ostreatus* was precultured on potato dextrose agar (PDA) and incubated at 25°C until mycelia filled the plate. The precultured *P. ostreatus* strain was kept at 4°C as a stock culture. *P. ostreatus* mushroom spawn was generated using distilled water-soaked bird seed grains. Four grams of wet bird seed grains were autoclaved with 1 g gypsum and 300 g calcium carbonate. The grains were infected aseptically with previously produced mushroom cultures and incubated at 25°C until colonised by mycelia [4]. The prepared mushroom spawn was kept at 4°C. Local substrates (sugarcane bagasse and sugar cane tops) were speckled with H_2_O to 65% moisture. Substrates were then supplemented with 20% wheat bran. These supplement doses were completely mixed with the substrates and then pasteurised at 60–65°C for six hours. Pasteurized substrates were infected with prepared spawn and incubated until mycelia colonised them. Once substrates were colonised with mushroom mycelia, they were transported to a 30% shade cloth fruiting chamber. *P. ostreatus* mushrooms fruited under ambient temperatures with continual fogging to reach 60% moisture, perfect for oyster mushrooms.

Mushroom extract was prepared using a modified technique. Freshly collected mushrooms were sun-dried beneath a 30% shade tunnel. Using a Scientec hammer mill, the dried mushrooms were ground into a 2 mm mesh powder. A 100 g of mushroom powder was dissolved in 250 ml methanol and incubated at 25°C for 24 h. The mushroom extract was filtered and evaporated using fume mode. Semidry *P. ostreatus* mushroom extract was then kept at 4°C [4].

### 2.3. Determination of Antimicrobial Activities of Extracts of Wild Mushrooms

The minimum inhibitory concentration (MIC) and minimum bacteriostatic concentration (MBC) of the extracts were obtained using the tetrazolium microtiter plate bioassay technique, which was then used to assess whether or not the extracts from wild mushrooms have antimicrobial properties. In order to make the stock solution of each mushroom extract, 10 mg/mL of the extract was dissolved in methanol. The extracts were classified based on the extraction substrate used, with each method being referred to by its abbreviation (*A*—20% Wheat Bran Sugar Cane, *B*—20% Wheat Bran Bagasse, *C*—20% Maize Flour Sugar Cane, and *D*—20% Maize Flour Bagasse), and the nanoparticles of zinc oxide being labelled as NPS.

At first, 90 *μ*l of Mueller-Hinton broth was put into each of the microtiter plate's 96 wells, which were arranged in eight rows and six columns. After that, 10 *μ*l of mushroom extract was put to Well #1 in Row A, which already contained the broth. The contents of Well #1 were then mixed and diluted in successive dilutions through Well #7, which produced concentrations ranging from 1 mg/mL (for Well #1) to 1 × 10^−6^ mg/mL (for Well #7), and on completion of the serial dilution, 10 *μ*l of content from well seven was discarded leaving the final volume in all the Wells at 90 *μ*l. Three rows (Well #8–#10) were labelled as “positive control,” “negative control,” and “quality control,” respectively, for each organism investigated. Positive control included *N. gonorrhoeae* (ATCC 49926) and no extract whereas the negative control contained only broth and no extract or organism, and the quality control contained the broth and the extract only.

After covering the microtiter plate with parafilm and placing it in a 5% CO2 incubator at a temperature of 37°C for 24 h. Following an incubation period of 24 h, the minimum inhibitory concentration (MIC) of each organism put to the test was determined by adding 5 *μ*l of an indicator dye containing 0.2 mg/mL of 2, 3, 5-triphenyltetrazolium chloride (TTC) into each well of the microtiter plate. After that, the microtiter plate was incubated at 37°C in a CO_2_ environment for three hours. At regular 30-minute intervals over the course of three hours, the TTC was inspected for any colour shifts. TTC is converted by biologically active bacterial cells into a colourless form of salt or a substance that is pinkish-red in colour. In cases where the colour of the broth and solutions in the well did not change while, we deduced that the bacteria had been suppressed. In order to measure the MBC of the contents, a loopful of inoculum was taken from each well of the microtiter plate and streaked over Chocolate Agar. At a temperature of 37°C and for a period of 24 h, the bacterial plates were kept in the CO_2_ incubator. The MBCs were then calculated as the lowest concentration of an antibacterial agent necessary to kill a bacterium over a specified period of time (18 to 24 hours), under the previously mentioned conditions.

## 3. Results

In this study, the antibacterial activities of *P. ostreatus* mushroom extracts were examined against target microorganisms. The mushroom extracts were prepared in one of four distinct methods. The isolates were initially put through a series of tests to find out whether or not they displayed any signs of antibiotic resistance, and their individual MICs were determined. Penicillin G, sulphonamides, ciprofloxacin, azithromycin, and ceftriaxone are the five known antibiotics that were used in the testing to determine whether or not the target *N. gonorrhoeae* isolates exhibited any signs of resistance to these antibiotics. Antibiotic discs were used for the preliminary screening, and the Etest was used to determine the minimum inhibitory concentration (MIC) for each antibiotic (see Table 1).

Only one of the isolates, identified by the number ISID 26, displayed any evidence of resistance to azithromycin; the minimum inhibitory concentration (MIC) for this isolate was noted at 1 g/ml. All of the isolates tested positive for sensitivity to ceftriaxone, and the majority of drug resistance was identified to be against two antibiotics, namely, sulphonamide and ciprofloxacin. Fifty percent of the isolates were able to impart an absolute resistance to both of these antibiotic agents. In addition, 40% of the isolates showed no sensitivity at all to the antibiotic penicillin G. All other isolates showed exhibiting signs of growing resistance to penicillin G, sulphonamide, and ciprofloxacin, with minimum inhibitory concentrations (MICs) ranging from 0.125 to 24 g/ml.

### 3.1. *P. ostreatus* Mushroom Extracts

The current investigation has shown that mushroom extracts possess antimicrobial properties.  Table 2 displays the results of the MBC performed on each individual bacterium.

**Figure 1:**
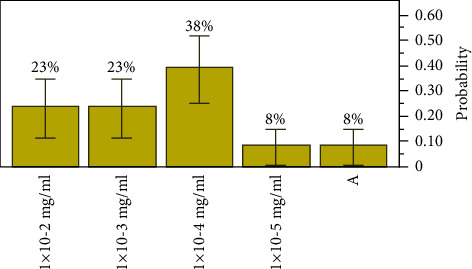
Distributions and test probabilities of extract *A* on target organisms.

The extract concentration, for extract *A* (see Figure 1), the lowest concentration of the antibacterial agent required to inhibit the target bacteria, was seen between 1 × 10^−2^ mg/ml of an estimated probability of 0.23077, a 0.081795 lower 95% confidence interval (95% CI), and an upper 95% CI of 0.502564; and 1 × 10^−5^ mg/ml of an estimated probability of 0.07692, a 0.01371 lower 95% CI, and a 0.33314 upper 95% CI. The most inhibitory concentration was observed at 1 × 10^−4^ mg/ml, *p*=0.38462, with an MBC elimination rate of 38% of the all-target organisms.

**Figure 2:**
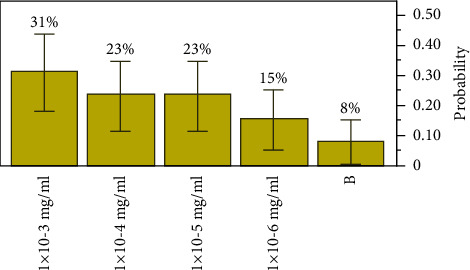
Distributions and test probabilities of extract *B* on target organisms.

Extract *B* exhibited the lowest concentration of the antibacterial agent required to inhibit the target bacteria between 1 × 10^−3^ mg/ml (*p*=0.30769, a lower 95% CI of 0.126807 and an upper 95% CI of 0.576307) and 1 × 10^−6^ mg/ml (*p*=0.15385, a lower 95% CI of 0.043258, an upper 95% CI of 0.422346). The most inhibitory concentration was observed at 1 × 10^−3^ mg/ml, with an MBC elimination rate of 31% of the all-target organisms, as shown in Figure 2.

**Figure 3:**
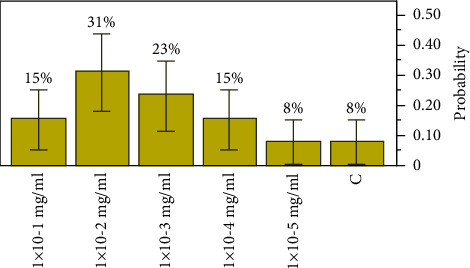
Distributions and test probabilities of extract *C* on target organisms.

For the extract concentration, of extract *C*, the lowest concentration of the antibacterial agent required to inhibit the target bacteria was seen between a wider range of 1 × 10^−1^ mg/ml (*p*=0.15385, a 0.043258 lower 95% CI and a 0.422346 upper 95% CI) and 1 × 10^−5^ mg/ml (*p*=0.07692, a 0.01371 lower 95% CI and a 0.33314 upper 95% CI). The most inhibitory concentration was observed at 1 × 10^−2^ mg/ml, *p*=0.30769, with an MBC elimination rate of 31% of the all-target organisms, as shown in Figure 3.

**Figure 4:**
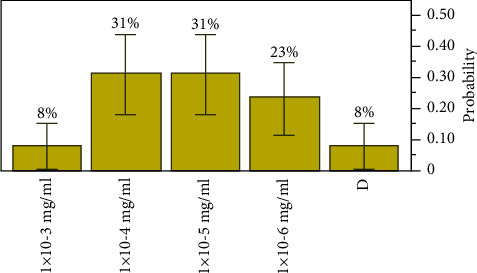
Distributions and test probabilities of extract *D* on target organisms.

The extract concentration, of extract *D*, the lowest concentration of the antibacterial agent required to inhibit the target bacteria, was seen between the range of 1 × 10^−3^ mg/ml with an estimated *p*=0.07692, a lower 95% CI of 0.01371, and an upper 95% CI of 0.33314 and 1 × 10^−6^ mg/ml with an estimated *p*=0.23077, a lower 95% CI of 0.081795, and an upper 95% CI of 0.502564. The most inhibitory concentration was observed at 1 × 10^−4^ mg/ml and 1 × 10^−5^ mg/ml, *p*=0.30769 with an MBC elimination rate of 31% of the all-target organisms, respectively, as shown in Figure 4.

**Figure 5:**
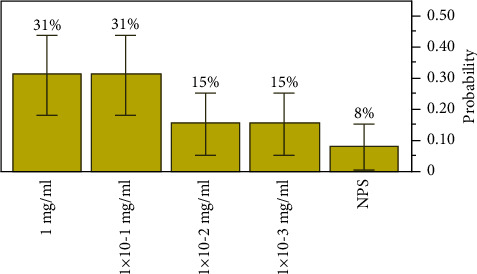
Distributions and test probabilities of extract NPS on target organisms.

The extract concentration, of extract NPS, the lowest concentration of the antibacterial agent required to inhibit the target bacteria, was seen between the range of 1 mg/ml (*p*=0.30769, a lower 95% CI of 0.126807 and an upper 95% CI of 0.576307) and 1 × 10^−3^ mg/ml with an estimated probability of 0.15385, a lower 95% CI of 0.043258, and an upper 95% CI of 0.422346. The most inhibitory concentration was observed at 1 mg/ml and 1 × 10^−1^ mg/ml, *p*=0.30769 with an MBC elimination rate of 31% of the all-target organisms, respectively, as shown in Figure 5.

All these, the 100 (1-*α*)% confidence interval including the true value of the population parameter with probability was seen at 0.950. From all the clinical isolates investigated, ISID 26 and 55 exhibited the most resistance according to MIC and MBC across all extracts, with a mean MBC of approximately 1 × 10^−2^ mg/ml, respectively.

## 4. Discussion

Multidrug-resistant organisms are becoming more prevalent and affecting the treatment of an expanding range of infectious illnesses [34]. As a consequence, there is an urgent need for the discovery of novel and efficient antimicrobials to combat pathogens now resistant to antibiotics [35]. It has been shown that fungi are excellent potential sources of bioactive chemicals with significant medicinal value. In addition, they are the most abundant sources of secondary metabolites. This article discusses mushroom extracts that have shown potential antibacterial activity against the target bacteria, namely ATCC strains of *S. aureus*, *E. coli*, *N. gonorrhoeae,* and nine other clinical *N. gonorrhoeae* isolates. Extracts *B* and *D* of *P. ostreatus* containing 20% wheat bran bagasse and 20% maize flour bagasse, respectively, exhibited outstanding antibacterial activity against *S. aureus, E. coli*, and all *N. gonorrhoeae* isolates investigated. Regarding the choice of extraction medium, bagasse extractions produced extracts with superior antibacterial activity against all organisms involved in this study. Phenolic compounds occur in plants as glycosides or aglycones, and owing to variations in stability, they may also exist as matrix and free-bound compounds [36]. Differences in structure may also alter the presence of phenolic compounds, causing them to exist as polymerized or monomers. Phenolic chemicals are not uniformly distributed in plants, and their stability varies, making extraction more difficult. The recovery rate of phenolic chemicals from samples may be influenced by single-step extraction and ineffective extraction procedures [23, 37]. As a result, selecting an appropriate extraction process is critical for extracting the desired phenolic compounds. Extraction of these compounds can be achieved by using conventional extraction, ultrasonic-assisted extraction (UAE), reflux extraction, microwave-assisted extraction (MAE), Soxhlet extraction, supercritical fluid extraction (SFE), and pulsed electric field extraction (PEF) [36]. During the extraction of sugar from sugarcane, a residual biomass known as bagasse is produced equal to around 25% of the total sugarcane processed. Bagasse contains a high calorific value, and of recently, bagasse has been utilised to produce biogas since it includes hemicellulose, cellulose, lignin, and soluble sugars. However, the production of biogas from bagasse is not profitable owing to its stubborn nature; as a result, several pretreatment procedures are being investigated to reduce the recalcitrance of the lignin-protected substrate. Our results imply that the presence of bagasse in mushroom extractions leads to increased extraction of bioactive chemicals, resulting in increased antibacterial activity against the at least three bacterial species, which were investigated in the current study. In the presence of 20% wheat bran bagasse and 20% maize flour bagasse, mushroom extracts had a greater concentration of bioactive compounds than extracts with 20% wheat bran sugarcane and 20% maize flour sugarcane. In this context, our results are comparable to those of recent research on the antibacterial effects of local mushrooms on other human pathogens.

Many antimicrobial phenolic chemicals, including as p-OH benzoic acid, p-OH-phenylacetic acid, protocatechuic acid, syringic acid, to name a few, may be produced during the extraction [24]. This is consistent with the results of a research on the antibacterial properties of the whole fruiting bodies of *P. ostreatus* against various common fungi study, which discovered that both fruiting bodies and mycelia derived from in vitro cultures may produce compounds with potential medical, pharmacological, and cosmetic applications [2, 12]. Antimicrobial properties of wild mushroom extracts derived from various solvents have also been demonstrated by other studies. In line with our findings, methanolic, ethanolic, and aqueous of *P. ostreatus* extracts were reported to have an antibacterial effect on the growth of *E. coli* cultures [4, 28]. Additionally, a different study that evaluated the antibacterial properties of *P. ostreatus* extracts against *S. aureus* reported that petroleum ether and acetone extracts of *P. ostreatus* were found to be effective against *Staphylococcus* spp. [28]. We thus propose that the variable antibacterial activity of *P. ostreatus* against *S. aureus* is attributable to changes in the geographical locations of the mushroom's habitats and the substrates used extraction [4, 5, 28]. A recent study compared the antibacterial properties of mushroom extract efficacies between Gram-negative and Gram-positive bacteria. It was concluded that the extracts were more effective in inhibiting Gram-negative bacteria than Gram-positive bacteria. The difference in sensitivity to antibiotic extracts between Gram-positive and Gram-negative bacteria has been linked to morphological variations in multiple studies [12, 38, 39]. It is suggested that the outer membrane of Gram-negative bacteria contains lipopolysaccharide, rendering the cell wall impervious to lipophilic extracts [40]. This paves the way for more research into the currently investigated *P. ostreatus* extracts against a wider range of Gram-positive and Gram-negative bacteria. In the current study, no statistically significant difference between Gram-positive and Gram-negative organisms was observed.

Literature has shown that various mushroom extracts and different extraction techniques have diverse antibacterial properties against Gram-positive and Gram-negative bacteria [12, 13]. Our results concur with research on the antibacterial properties and mineral compositions of various mushrooms grown on agricultural waste. In the case of the present study, we have shown that *S. aureus*, a Gram-positive bacterium, is extremely sensitive and inhibited 20% wheat bran bagasse extractions compared to all *N. gonorrhoeae* clinical isolates and other ATCC strains included in this investigation. This is corroborated by recent research demonstrating that both chloroform and water extracts of wild mushrooms had potent antibacterial properties against *S. aureus* than other Gram-negative bacteria investigated. Focusing on the clinical isolates, the 20% wheat bran bagasse extracts exhibited the most potent antibacterial activity against the *N. gonorrhoeae* organisms, and when compared to the antibacterial activities of the five antibiotics also investigated in this study, it was observed that the two isolates, ISID 26 and ISID 55, that exhibited the greatest resistance to the five antibiotics during screening had the highest MIC and MBC when compared to other target clinical isolates. These findings indicate that the concentration of *P. ostreatus* extract needed to effectively suppress the growth of a specific microorganism increases proportionally with the antimicrobial resistance and spectrum of that organism. The antibacterial activity of *P. ostreatus* extracts examined in this research varied to variable degrees within the same species of organisms. Variation in the antibacterial activity of the extracts may be attributable to resistance factors such as conjugative plasmids, transposons, and insertion sequences. Furthermore, we postulated that the differences in antimicrobial activities may be attributable to a variety of factors, including the genetic configurations of the clinical isolates, which may result in alterations of physical and biochemical nature of the organism due to mutations or this may be due to the substrate used in the extraction of bioactive agents. Due to the efficacy of the extraction technique, the lack of substantial secondary metabolites might account for the decreased antibacterial activity of the 20% Wheat Bran Sugar Cane and 20% Maize Flour Sugar Cane extracts. The extracts have resulted in varied in MIC antimicrobial activities against *E. coli* and *S. aureus* with a mean extract concentration of 1 × 10^−5^ mg/mL, whereas the *N. gonorrhoeae* ATCC 49926 had an MIC mean concentration of 1 × 10^−3^ mg/mL, suggesting that *N. gonorrhoeae* possess a greater resistance to the target extracts when compared to *E. coli* and *S. aureus*.

The antibacterial activity of all the extracts examined in the current investigation has displayed some level of efficacy against the clinical isolates and standard strains. However, the majority of clinically isolated bacteria have shown more resistance to the extracts than the standard *N. gonorrhoeae* strain. The primary cause is the indiscriminate exposure of clinical isolates to multiple antimicrobial drugs.

## 5. Conclusion

The current study demonstrates that polyphenols are a prospective source of efficient, secure, and cost-effective antimicrobial compounds. The antimicrobial potential of natural compounds presents a broad spectrum of options for novel antibacterial therapeutics against multidrug-resistant clinical isolates, despite the fact that this investigation solely focused on in vitro analysis. Due to their inadequate therapeutic impact, polyphenols with MICs greater than existing antibiotics cannot be used in antimicrobial monotherapy; nevertheless, the use of antibiotics in combination treatment may enhance the pharmacokinetic and pharmacodynamic characteristics of these polyphenols. Additionally, using polyphenols can make it possible to lower medicine doses and lessen antibiotic adverse effects. To determine the utility of these antibacterial drugs in the therapeutic setting, more research should concentrate on in vivo experiments and clinical trials.

## Figures and Tables

**Table 1:** Antibiotic discs preliminary screening, as well as the determination of the minimum inhibitory concentration (MIC), performed on each isolate against known antibiotics.

	Disc diffusion test	Etest	Disc diffusion test	Etest	Disc diffusion test	Etest	Disc diffusion test	Etest	Disc diffusion test	Etest

ISID (isolate ID)	Pen G 1.5 iu	Pen G (0.002–32 *μ*g/ml)	Sulph	Sulph (0.002–32 *μ*g/ml)	Cipro	Cipro (0.002–32 *μ*g/ml)	Azythro	Azythro (0.016–256 *μ*g/ml	Ceft 30 mcg	Ceft (0.002–32 *μ*g/ml)
			300 mcg		5 mcg		15 mcg			
4	G–R	0.75	G–R	0.125	R	>32	S	0.032	S	0.008
5	R	>32	R	>32	R	>32	S	<0.016	S	0.002
7	R	>32	R	>32	R	>32	S	0.047	S	0.12
17	G–R	12	R	>32	G–R	6	S	0.094	S	0.004
26	G–R	8	R	>32	G–R	3	G–R	1	S	0.004
28	G–R	6	G–R	16	G–R	8	S	0.023	S	<0.002
39	G–R	12	G–R	16	G–R	24	S	0.064	S	0.008
45	R	>32	R	>32	R	>32	S	<0.016	S	0.003
55	G–R	2	G–R	1.9	R	>32	S	0.32	S	<0.002
ATCC 49226	R	>32	R	>32	G–R	0.5	S	<0.016	S	0.004

G–R = gaining resistance, R = resistant, S = susceptible.

**Table 2:** MBC determined for *P. ostreatus* mushroom extracts antimicrobial properties against target bacteria.

*P. ostreatus* mushroom extracts					
Isolate ID	*A*	*B*	*C*	*D*	NPS
*S. aureus* (ATCC 25923)	1 × 10^−3^ mg/ml	1 × 10^−6^ mg/ml	1 × 10^−1^ mg/ml	1 × 10^−5^ mg/ml	1 × 10^−4^ mg/ml
*E. coli* (ATCC 25922)	1 × 10^−2^ mg/ml	1 × 10^−4^ mg/ml	1 × 10^−3^ mg/ml	1 × 10^−5^ mg/ml	1 × 10^−5^ mg/ml
*N. gonorrhoeae* (ATCC 49926)	1 × 10^−3^ mg/ml	1 × 10^−5^ mg/ml	1 × 10^−2^ mg/ml	1 × 10^−4^ mg/ml	1 mg/ml
ISID 4	1 × 10^−3^ mg/ml	1 × 10^−3^ mg/ml	1 × 10^−4^ mg/ml	1 × 10^−6^ mg/ml	1 × 10^−3^ mg/ml
ISID 5	1 × 10^−3^ mg/ml	1 × 10^−5^ mg/ml	1 × 10^−2^ mg/ml	1 × 10^−5^ mg/ml	1 × 10^−4^ mg/ml
ISID 7	1 × 10^−2^ mg/ml	1 × 10^−5^ mg/ml	1 × 10^−1^ mg/ml	1 × 10^−6^ mg/ml	1 × 10^−2^ mg/ml
ISID 17	1 × 10^−4^ mg/ml	1 × 10^−6^ mg/ml	1 × 10^−3^ mg/ml	1 × 10^−4^ mg/ml	1 × 10^−3^ mg/ml
ISID 26	1 × 10^−2^ mg/ml	1 × 10^−3^ mg/ml	1 × 10^−2^ mg/ml	1 × 10^−4^ mg/ml	1 mg/ml
ISID 28	1 × 10^−4^ mg/ml	1 × 10^−3^ mg/ml	1 × 10^−5^ mg/ml	1 × 10^−4^ mg/ml	1 × 10^−1^ mg/ml
ISID 39	1 × 10^−4^ mg/ml	1 × 10^−4^ mg/ml	1 × 10^−3^ mg/ml	1 × 10^−5^ mg/ml	1 × 10^−2^ mg/ml
ISID 45	1 × 10^−3^ mg/ml	1 × 10^−4^ mg/ml	1 × 10^−4^ mg/ml	1 × 10^−6^ mg/ml	1 mg/ml
ISID 55	1 × 10^−2^ mg/ml	1 × 10^−3^ mg/ml	1 × 10^−2^ mg/ml	1 × 10^−3^ mg/ml	1 × 10^−3^ mg/ml

*P. ostreatus* extracts revealed varying degrees of antibacterial activity against the pathogens examined (Table 2). The majority of clinical samples were shown to be most susceptible to extracts *B* and *D* and least susceptible to extracts *C* and NPS.

## Data Availability

The data supporting the current study are available from the corresponding author upon request.
